# Perioperative poly(I:C) reverses accelerated tumor growth after surgery in neuroblastoma

**DOI:** 10.1093/immhor/vlaf058

**Published:** 2025-10-09

**Authors:** Chenkai Mao, Maria Poimenidou, Donna McAllister, Michael B Dwinell, Brian T Craig

**Affiliations:** Department of Surgery, Medical College of Wisconsin, Milwaukee, WI 53226, United States; Center for Immunology, Medical College of Wisconsin, Milwaukee, WI 53226, United States; Center for Immunology, Medical College of Wisconsin, Milwaukee, WI 53226, United States; Department of Microbiology & Immunology, Medical College of Wisconsin, Milwaukee, WI 53226, United States; Center for Immunology, Medical College of Wisconsin, Milwaukee, WI 53226, United States; Department of Microbiology & Immunology, Medical College of Wisconsin, Milwaukee, WI 53226, United States; Department of Surgery, Medical College of Wisconsin, Milwaukee, WI 53226, United States; Center for Immunology, Medical College of Wisconsin, Milwaukee, WI 53226, United States; Department of Microbiology & Immunology, Medical College of Wisconsin, Milwaukee, WI 53226, United States; Department of Surgery, Medical College of Wisconsin, Milwaukee, WI 53226, United States; Center for Immunology, Medical College of Wisconsin, Milwaukee, WI 53226, United States

**Keywords:** surgery, perioperative, high-risk neuroblastoma, poly(I:C), systemic immunity

## Abstract

Surgery for local control is a cornerstone of anticancer therapy with demonstrated survival benefit. However, surgery-induced modulation of antitumor immunity may also contribute to cancer progression and relapse. Despite evidence for a pro-tumor surgery effect in adult cancers, there remain significant knowledge gaps as to the influence surgery has on recurrence or metastatic outgrowth in pediatric cancers. High-risk neuroblastoma (HR-NB) is the most common extracranial solid tumor of childhood. While almost all children with HR-NB undergo surgery, nearly 50% suffer metastatic relapse and succumb to their disease. To ascertain if surgery may contribute to recurrence in HR-NB, we developed a mouse model to comprehensively interrogate the systemic effect of surgery on distant tumor growth and immune modulation. This model demonstrated that *MYCN*-amplified HR-NB tumor growth was accelerated by surgery compared to tumor-bearing mice without surgical stress. Accelerated tumor growth was absent in HR-NB cells engrafted to immune deficient mice, suggesting that an intact immune system may be needed for surgery to exert its pro-growth effect on distant tumor cells. Consistent with that genetic ablation model, flow cytometry measured a decrease in splenic macrophages (Mϕ) and dendritic cells (DC) and an increase in myeloid-derived suppressor cells (MDSC) after surgery. Perioperative treatment with polyinosinic-polycytidylic acid [poly(I:C)] ameliorated surgery-induced tumor growth. These findings provide direct insight into the systemic surgical effect on pediatric solid tumor growth and identify innate immune adjuvants as a potential perioperative treatment to mitigate this effect.

## Introduction

Surgery is a critical component in the clinical management of malignant solid tumors. Despite clear therapeutic benefit from surgical removal of tumors,^1^ it has long been thought that components of the host response to surgery may contribute to residual disease progression.^2^ Halstead’s experience with mastectomy for breast cancer at the turn of the twentieth century was one of the first observations that surgery led to accelerated metastatic progression.^3^ Much more recently, adoption of laparoscopy for abdominal cancer surgery demonstrated that lowering the “dose,” or magnitude, of surgery for colon cancer improved stage-specific survival compared to open surgery.^4^ Pre-clinical rodent models have been established that demonstrate a pro-metastatic effect for surgery in melanoma,^5^ breast,^6^ and colon^7^ adenocarcinoma, and osteosarcoma.^8^ Multiple facets of tumor and host response potentially contribute to the observed pro-tumorigenic effect of surgery.^9^

The immune system serves as a primary response system for any form of physical injury, including surgery.^10^ Altered immune cellular influx to the metastatic tumor microenvironment as well as impaired immune cellular function has been reported in the context of surgery for cancer. For example, surgery increases the influx of neutrophils and the elaboration of neutrophil extracellular traps in the liver after resection of metastatic colon cancer, which subsequently facilitated tumor growth and increased liver metastatic burden.^11^ Natural killer (NK) cells demonstrated compromised cytokine production after surgery in colorectal cancer.^12^ In a lung cancer model, tumor resection led to increased levels of pro-tumorigenic cytokines such as vascular endothelial growth factor (VEGF), interleukin 6 (IL-6), and a decrease of interferon-γ (IFN-γ);^13^ MDSC were increased after thoracotomy and promoted angiogenesis and tumor growth.^14^ These established cellular immune changes with surgery suggest that altered immunity may be a primary mechanism of the pro-tumorigenic surgery effect.

A unique aspect of the clinical care for pediatric solid tumors is the deployment of surgery for local control of the primary tumor despite the presence of known metastatic disease.^15^ Nearly all children with metastatic neuroblastoma, the most common pediatric extracranial solid tumor, undergo a major abdominal operation for local control.^16^ Relapse and progression rates hover near 50% despite major advancements in systemic therapy in the past 20 y.^17^ Identifying all factors that lead to progression, relapse, and treatment resistance is a major priority for research.^18^ We hypothesize that patient response to surgery may be one factor contributing to disease progression and relapse and may present a novel therapeutic target with immune-modifying agents. Critically, a pre-clinical model system of the effect of surgery in neuroblastoma has yet to be reported.

In this study, we investigated (1) whether there is a systemic host effect elicited by surgery that is specific to the non-excisional components of surgery and (2) whether this systemic effect is immune mediated. Furthermore, we were interested in testing whether perioperative innate immune activation could reverse the surgery effect. Our data demonstrated that the non-excisional components of surgery promote distant tumor outgrowth and modulate systemic immunity. Innate immune activation with perioperative administration of poly(I:C) reversed the surgery-induced pro-growth effect.

## Materials and methods

### Cell culture

9464D is a murine neuroblastoma cell line derived from spontaneously occurring *MYCN-*amplified neuroblastoma tumors in the transgenic Th-*MYCN* mouse model on a C57BL/6 background.^19^ 9464D cells were a generous gift from Dr. Rimas Orentas (Seattle Children’s Hospital, Seattle, Washington, USA). B16F10 melanoma cells and MC38 colon adenocarcinoma cells were purchased from the American Type Culture Collection (Manassas, Virginia, USA). Cells were maintained in Dulbecco’s Modified Eagle Medium (DMEM; Gibco, Life Technologies), supplemented with 10% heat-inactivated fetal bovine serum (Omega Scientific, Inc.) and 2 mM L-glutamine at 37°C and 5% CO_2_. Cells were harvested/passaged at 80% confluency using TrypLE Express (Gibco, Life Technologies).

### Animal experiments

Male and female wild-type C57BL/6J mice and *Rag2^(−/−)^γc^(−/−)^Cd47^(−/−)^* (“triple knockout”; TKO) mice on C57BL/6J background were purchased from the Jackson Laboratory (JAX, Bar Harbor, Maine, USA) and bred in house. All mice were age matched (6–10 wk old) at the beginning of each experiment. All animal studies were approved in advance by the Institutional Animal Care and Use Committee of the Medical College of Wisconsin under approved protocol no. 7531. Mice were anesthetized with 2% to 3% inhaled isoflurane and then 2 × 10^6^ 9464D, 3 × 10^5^ B16F10, or 3 × 10^5^ MC38 tumor cells in 50 µl phosphate-buffered saline (PBS) were injected intradermally over the right flank. Shortly after implantation, mice were randomized to receive a 12-min surgical procedure (described below) or no surgery. Tumor growth was tracked by serial caliper measurements of longest dimension length (*l*) and shortest dimension width (*w*) every other day until the humane endpoint for tumor size was reached per institutional policy. For treatment with poly(I:C) (InvivoGen), 150 µg of poly(I:C) in 100 µl PBS was injected intraperitoneally (IP) beginning 1 d prior to surgery and tumor implantation, continuing every 5 d until the endpoint. Tumor volume was calculated using the formula *l × w × w*/2. Growth curves stop whenever the first mouse in the group reaches the IACUC-approved humane tumor volume endpoint of 500 mm^3^, and this is reported as tumor growth. After this point, tumor-specific survival is evaluated as time to reach the humane endpoint for the remainder of the experimental cohort.

### Surgical procedure

Surgery occurred on the same day as tumor cell implantation. Mice were anesthetized with inhaled 2% to 3% isoflurane and maintained in a non-responsive plane of anesthesia using 1% to 2% isoflurane administered via a nose cone throughout the procedure. Buprenorphine-SR (1.5 mg/kg) was subcutaneously administered for analgesia during and after surgery. After shaving, the abdomen was prepped with a combination of chlorhexidine and ethanol. A midline incision was made in the skin and the peritoneal cavity was opened. A self-retaining retractor was used to ensure that the peritoneal cavity remained widely open. To promote evaporative fluid losses, intestines were exteriorized in a caudal direction. Direct tissue injury was induced by gently crushing the liver edge with a curved forceps. The peritoneal cavity remained open for an additional 4–6 min, before being closed in two layers with suture and surgical clips. Total experimental surgery times were 12.03 ± 0.86 min across all experiments (Fig. S1). Following surgery, mice were transferred to a recovery cage and placed on a 37°C warm water heating pad until they regained full activity levels, after which they were returned to their primary cage.

### Surface and intracellular staining of immune cells

Single-cell suspensions of mouse splenocytes and tumor-draining lymph node (TDLN) were prepared for flow cytometric analysis. Red blood cells in the spleen were lysed using ACK Lysis Buffer (Life Technologies). Fc receptors were blocked with anti-mouse CD16/32 (Biolegend) and immunostained with indicated markers as shown in Table S1. Foxp3/Transcription Factor staining buffer set was used for intracellular staining (eBioscience). We modified an established general gating strategy that enabled broad assessment of the myeloid and lymphoid compartment changes (Fig. S2).^20–22^ The lymphoid compartment changes were analyzed using the gating strategies as shown in Fig. S2A. Briefly, following gating to exclude doublets, debris and dead cells, NK cells were defined as CD45^+^/CD3^−^/NK1.1^+^. CD4^+^ T cells were identified as CD45^+^/CD3^+^/CD4^+^; CD8^+^ T cells were identified as CD45^+^/CD3^+^/CD8^+^. Based on the surface expression of CD25 and the intracellular staining of Foxp3, Treg were identified as CD45^+^/CD3^+^/CD4^+^/CD25^+^/Foxp3^+^. For myeloid populations, following gating as shown in Fig. S2B to exclude doublets, debris and dead cells, M-MDSC were defined as CD45^+^/CD3^−^/CD11b^+^/Ly6C^hi^, and PMN-MDSC were defined as CD45^+^/CD3^−^/CD11b^+^/Ly6G^hi^. Following the exclusion of MDSC, F4/80^+^ macrophages were identified by high F4/80 expression and DC cells were defined as F4/80^−^/CD11c^+^. A Cytek Aurora cytometer was utilized for all experiments and raw data processed and analyzed using SpectroFlo 1.0 software (Cytek Biosciences).

### Statistics and reproducibility

All results are expressed as mean ± standard error. Student's *t* test was used to compare the statistical differences between 2 groups. Tumor growth curves were analyzed using 2-way ANOVA given the repeated measures nature of this data. Survival rates were analyzed using the log-rank (Mantel-Cox) test. GraphPad Prism 10.1.1 software was used to test for differences between the samples and create visualizations. All experiments were repeated, and representative figures were presented unless otherwise noted. Exact calculated *P* values are reported. The statistical test utilized is indicated in each figure legend.

## Results

### Accelerated tumor growth after surgery (AGAS) in a murine model of neuroblastoma

**Figure 1.**
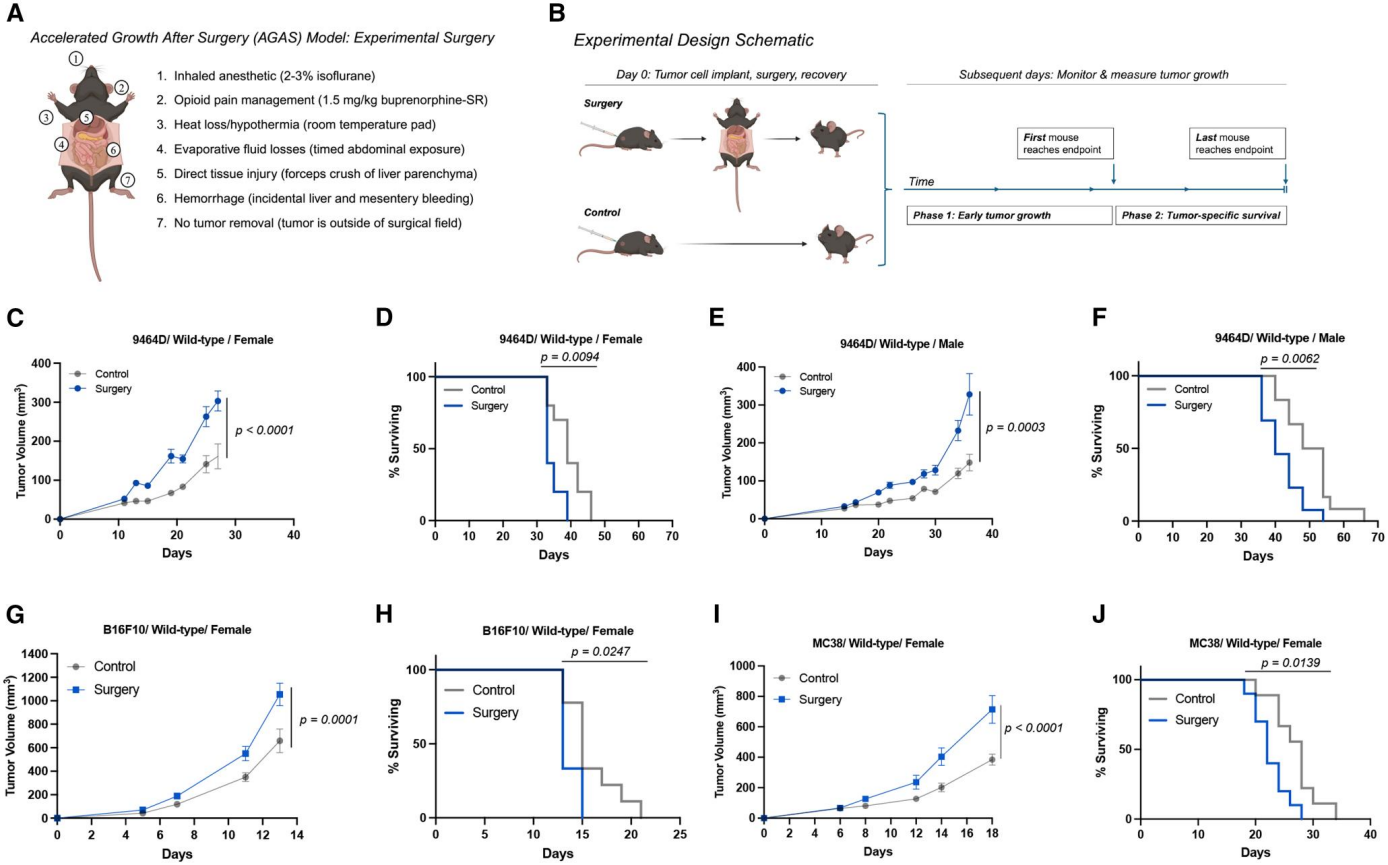
Surgery model, experimental design, and accelerated growth after surgery (AGAS) in mouse neuroblastoma. (A) Schematic of mouse abdominal surgery that involves sterile access of the peritoneal cavity via a midline incision, exteriorization of the intestines, and controlled forceps crush of the liver edge to model the physical collateral tissue injury encountered by a patient undergoing an abdominal neuroblastoma removal operation. (B) Schematic of the experimental design to test for surgery effect on distant tumor growth. Tumor progression is assessed in 2 phases. (C) Six-week-old female C57BL/6J wild-type mice with 9464D cells are assessed for tumor growth by repeated direct caliper measurement of flank tumors. (D) The same female mice are assessed for survival to the pre-designated endpoint (*n* = 10 mice/group; 2 replicates). (E, F) Male mice of the same age with 9464D cells are assessed for tumor growth and tumor-specific survival (*n* = 10 mice/group; 2 replicates). (G, H) C57BL/6J wild-type mice with B16F10 cells are assessed for tumor growth and tumor-specific survival (*n* = 10 mice/group; 3 biological replicates in female mice, 1 biological replicate in male mice). (I, J) C57BL/6J wild-type mice with MC38 cells are assessed for tumor growth and tumor-specific survival (*n* = 10 mice/group; 3 biological replicates in female mice, 1 biological replicate in male mice). Exact calculated *P* values are reported. Tumor growth is tested using repeated measures 2-way ANOVA. Tumor-specific survival is tested by log-rank (Mantel-Cox) test.

To model the impact of surgical exposure on distant minimal disease that is already present outside the operative field at the time of surgery, we tested an abdominal surgery model that is derived from previously reported similar models^23^ that is relevant to the clinical surgical care performed for local control in children with high-risk neuroblastoma.^24^ We utilized an established mouse model of high-risk neuroblastoma,^25^ in which mouse-derived 9464D neuroblastoma tumor cells are implanted in the flank dermis of syngeneic, wild-type C57BL/6J mice to form tumors that respond to immunotherapy. To also test the generalizability of our findings to other tumor contexts, we additionally utilized 2 other cancer cell lines syngeneic to wild-type C57BL/6J mice: B16F10 melanoma and MC38 colon cancer. Our surgery model isolates the major components of physiologic stress and tissue injury that occurs in the clinical setting during tumor removal operations (Fig. 1A): inhaled anesthetic, opioid pain medication, temperature dysregulation (hypothermia), fluid losses (bleeding and evaporative losses), and direct tissue injury; to model direct tissue injury, in addition to the opening of the abdomen and manipulation of the intestines, a controlled crush injury of the liver edge is performed with forceps. Mice recover from surgery and tumor growth is monitored over time (Fig. 1B). Importantly, our model does not involve tumor removal, thereby isolating the systemic host response to surgery from the effects of tumor debulking. The implanted tumor is physically separated from the operative site, enabling investigation of the systemic effects of surgery on distant disease that exists outside the operative field though is present at the time of surgery. Tumor progression is assessed in 2 phases. In phase 1, serial tumor volume measurements are obtained by direct caliper measurement and followed over time. Once a tumor in the first mouse reaches or exceeds the pre-designated humane endpoint (500 mm^3^), phase 1 concludes, and phase 2 commences. In phase 2, survival to the endpoint is measured for each mouse and analyzed by the Kaplan–Meier approach. Female mice exposed to surgery demonstrate both faster tumor growth (Fig. 1C), and shorter tumor-specific survival (Fig. 1D). Similarly, male mice demonstrate the same effects (Fig. 1E, F). Mice implanted with B16F10 melanoma cells (Fig. 1G, H) or with MC38 colon cancer cells (Fig. 1I, J) also demonstrated accelerated tumor growth and shorter tumor-specific survival.

### Loss of AGAS phenotype in mice genetically lacking mature T, B, NK, and innate lymphoid cell (ILC) populations

**Figure 2.**
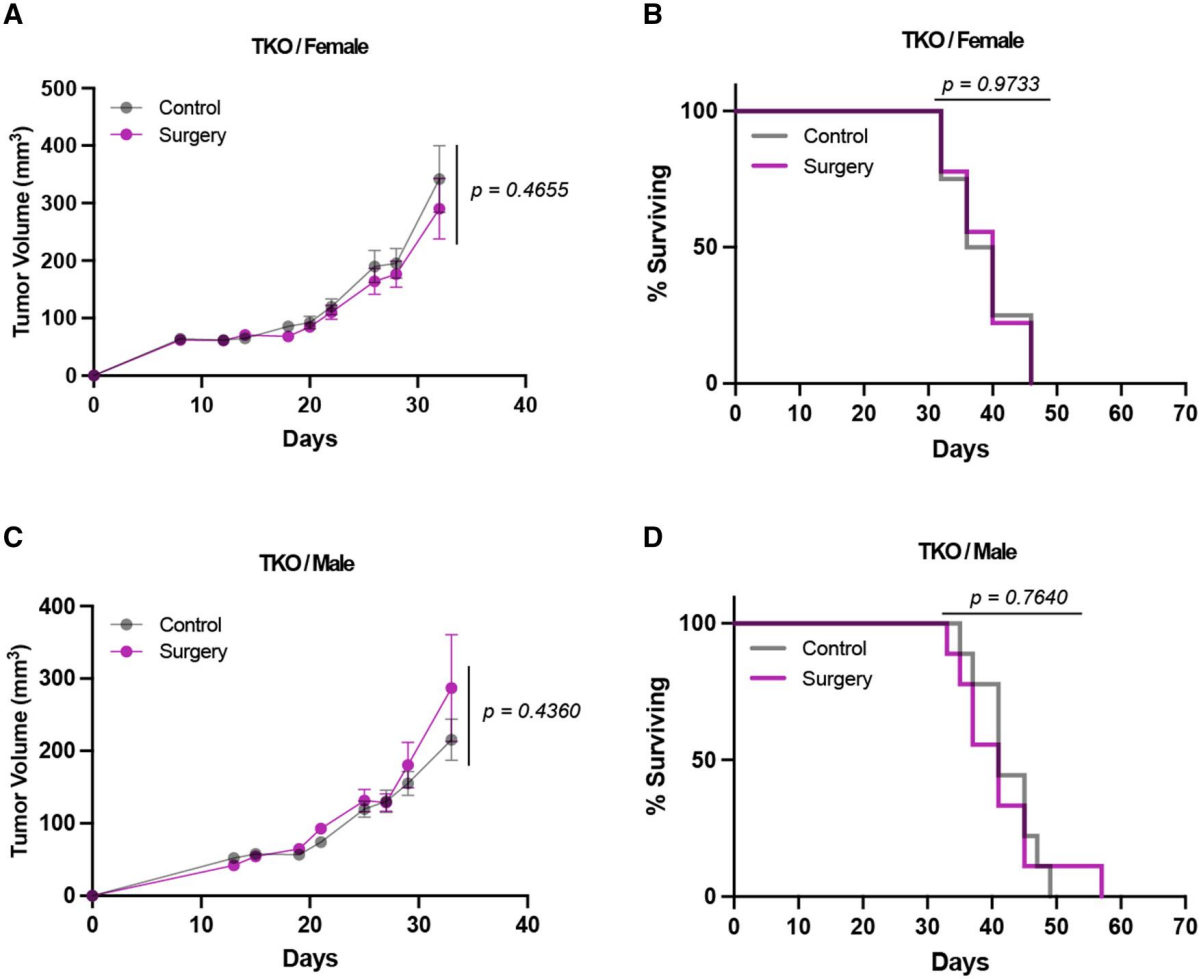
Loss of AGAS phenotype in mice genetically lacking T, B, and NK cells. “Triple knock out” (TKO) mice (B6.129S-*Rag2^tm1Fwa^ Cd47^tm1Fpl^ Il2rg^tm1Wjl^*/J) that are missing T, B, NK cells, and ILC are tested for AGAS. (A) Six-week-old female TKO mice are assessed for tumor growth by repeated direct caliper measurement of flank tumors. (B) The same female mice are assessed for survival to the pre-designated endpoint (*n* = 10 mice/group; 2 biological replicates). (C, D) Male mice of the same age are assessed for tumor growth and tumor-specific survival (*n* = 10 mice/group; 2 biological replicates). Exact calculated *p* values are reported. Tumor growth is tested using repeated measures 2-way ANOVA. Tumor-specific survival is tested by log-rank (Mantel-Cox) test.

We investigated whether AGAS is mediated by an immune mechanism by applying the same surgery model and HR-NB 9464D cell line to B6.129S-*Rag2*^tm1Fwa^*Cd47*^tm1Fpl^*Il2rg*^tm1Wjl/J^ (“triple knockout,” TKO) mice. These mice are maintained and validated by The Jackson Laboratory (Bar Harbor, Maine, USA). Mice are backcrossed for at least 10 generations onto C57BL/6J background and lack mature T, B, NK, and ILC cell populations due to their loss of *Rag2* and *Il2rg.*^26^^,^^27^ We confirmed the absence of mature T (CD4^+^ or CD8^+^), B, and NK cells by flow cytometry (Fig. S3). Like wild-type C57BL/6J mice, the 9464D cell line is also syngeneic with the TKO mouse. Female six-week-old TKO mice exposed to surgery demonstrated no differences in either tumor growth (Fig. 2A) or tumor-specific survival (Fig. 2B). Similarly, male TKO mice tested under the same conditions also demonstrated no differences in tumor growth (Fig. 2C) or tumor-specific survival (Fig. 2D). These data suggest that an intact immune system may be necessary for the AGAS effect.

### Increased immature myeloid cell frequency is coupled with decreased antigen presenting cell types early after surgery

**Figure 3.**
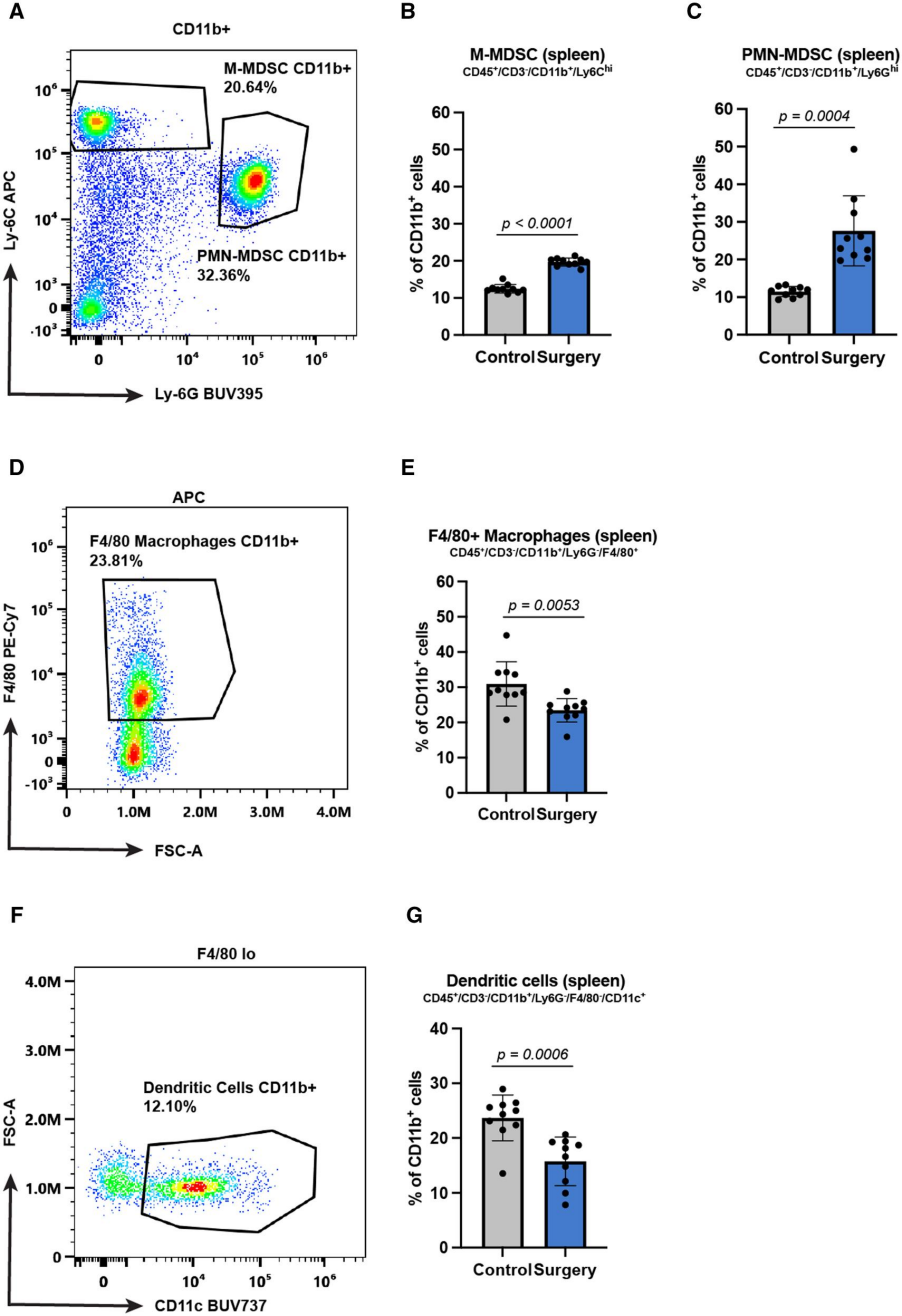
Systemic myeloid cell changes with surgery. Splenic myeloid cell populations were assessed by flow cytometry five days after surgery. (A–C) M-MDSC and PMN-MDSC are increased with surgery. (D, E) Total F4/80^+^ macrophages are decreased with surgery. (F, G) Total CD11c^+^ dendritic cell population are also decreased with surgery. Exact calculated *P* values are reported. Cell group comparisons were performed using *t* test with Welch’s correction. *n* = 10 female mice per group in all experiments with 2 biological replicates.

The loss of the AGAS phenotype in the absence of the major lymphocytic populations suggests that the AGAS effect is mediated through impaired systemic cellular immunity. To investigate this in an unbiased approach, we analyzed the systemic response of the major myeloid and lymphoid cell populations in the spleen 5 d after surgery, a time point that precedes the observed AGAS. Flow cytometric analysis was performed following an established gating strategy as described in the Materials and methods section.^20–22^ Immature myeloid precursors, termed myeloid-derived suppressor cells (MDSC), are known for their general pro-tumor and immunosuppressive functions.^28^ In particular, the monocytic subtype is associated with the immunosuppressive TME in solid tumors.^29^ Consistent with these reported observations, we detected an increase in both monocytic-MDSC (M-MDSC) and polymorphonuclear-MDSC (PMN-MDSC) subtypes with surgery exposure (Fig. 3A–C). Conversely, the F4/80^+^ macrophage population decreased after surgery (Fig. 3D, E). Similarly, dendritic cells were likewise decreased following surgery (Fig. 3F, G). In contrast, total CD3^+^, CD4^+^, and CD8^+^ T cell populations, regulatory T cells, and natural killer cells remained unchanged at this early time after surgery in spleen (Fig. S4A). On the other hand, we observed a subtle shift in the CD4^+^ and CD8^+^ T cells in TDLN between the surgery and control groups (Fig. S4B). That is, CD4^+^ T cells were decreased while CD8^+^ T cells were increased after surgery. Absolute cell counts for the major myeloid and lymphoid parent populations are listed in Tables S2–S5. No comparable myeloid analysis was conducted in TDLN due to the extremely low CD11b^+^ events as shown in Table S3.

### Perioperative application of the TLR3 agonist poly(I:C) reverses AGAS and modulates systemic myeloid cell response to surgery

**Figure 4.**
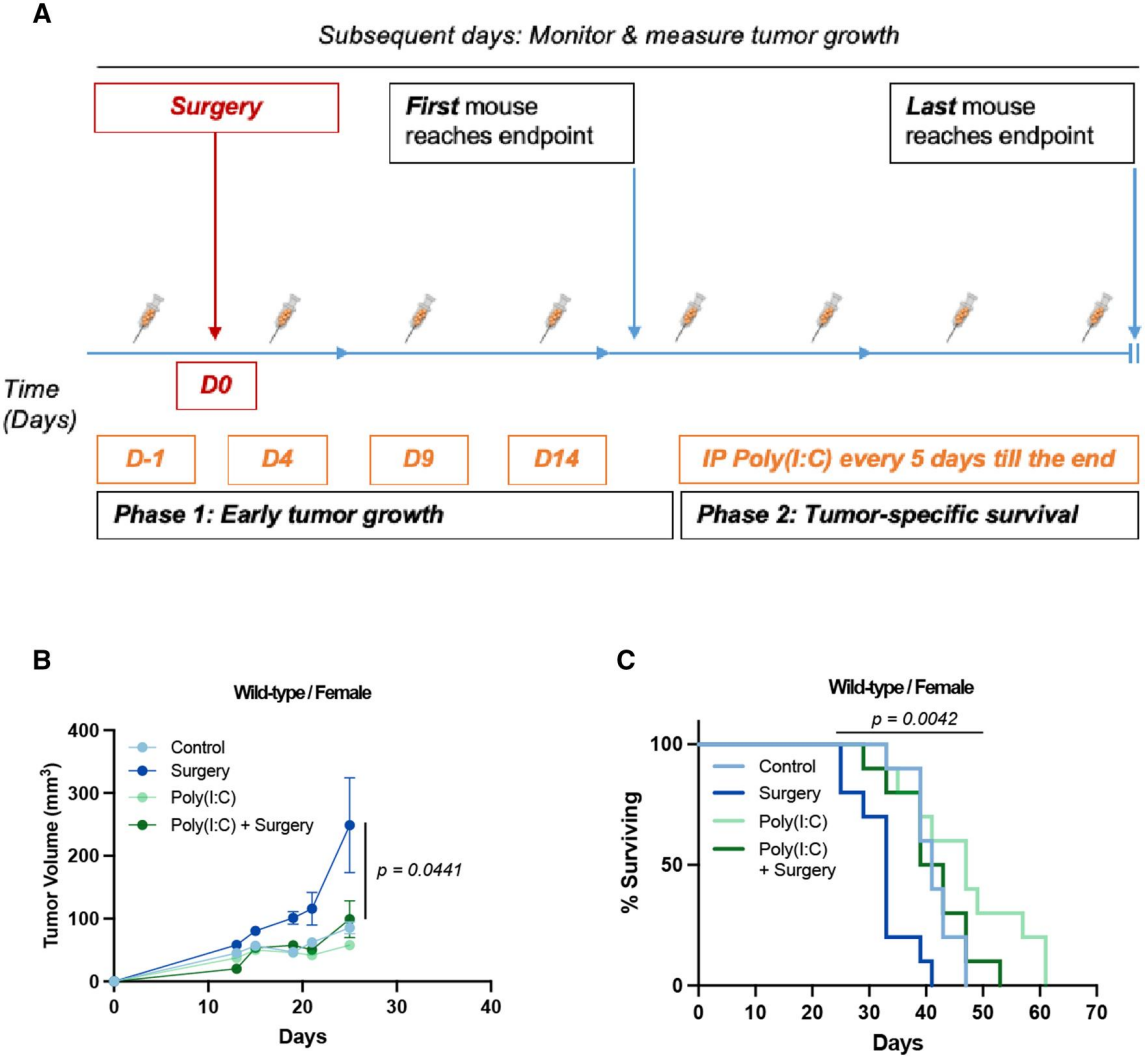
Perioperative poly(I:C) reverses AGAS. (A) Schematic of the experimental design with the timing of poly(IC) administration relative to surgery. Tumor progression is assessed in two phases. (B) Six-week-old wild-type C57BL/6J mice were administered 150 µg of poly(I:C) IP 1 d prior to tumor cell implantation and surgery exposure, and every 5 d until the endpoint was reached. Mice were assessed for tumor growth by serial direct caliper measurement. (C) The same mice were assessed for survival to the pre-designated endpoint. Exact calculated *P* values are reported (*n* = 10 mice/group; 2 biological replicates in female mice). Tumor growth is tested using repeated measures 2-way ANOVA. Tumor-specific survival is tested by log-rank (Mantel-Cox) test.

**Figure 5.**
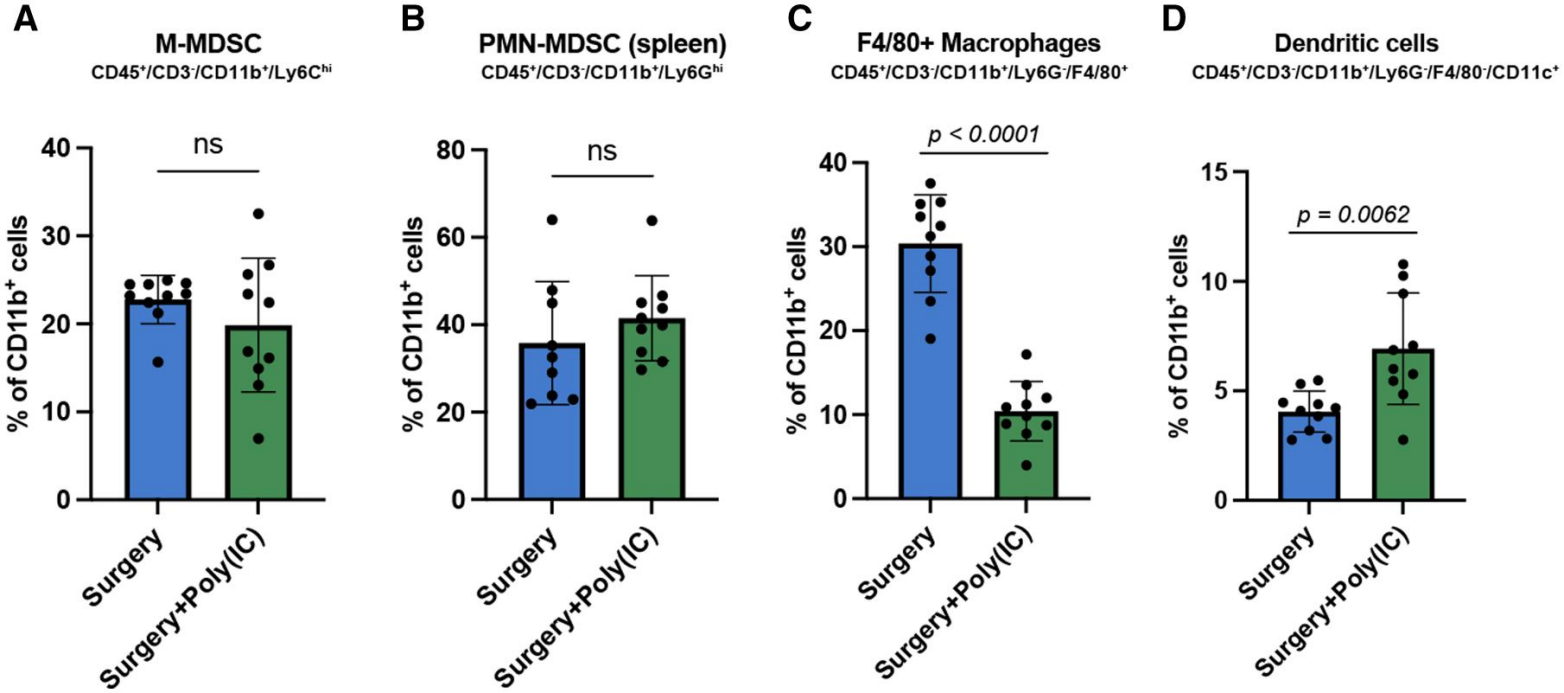
Poly(I:C) modulates the systemic myeloid response to surgery. Splenic myeloid cell populations were assessed by flow cytometry 5 d after surgery. (A, B) M-MDSC or PMN-MDSC is unaffected by poly(I:C) compared to surgery alone. (C) Total F4/80^+^ macrophages are markedly decreased with the addition of perioperative poly(I:C). (D) Total CD11c^+^ dendritic cell population is increased by poly(I:C). Exact calculated *p* values are reported. Cell group comparisons were performed using *t* test with Welch’s correction. *n* = 10 mice per group in all experiments with 2 biological replicates.

In previously reported models of surgery-accelerated tumor growth, poly(I:C), a known TLR3 agonist, and CpG-DNA, a known TLR9 agonist, have both demonstrated efficacy.^30^ Interestingly, systemic administration of poly(I:C) exhibited a sufficient therapeutic window whereby control animals did not show any signs of unwanted immune activation at doses that significantly slowed tumor growth.^31^ A derivative of poly(I:C), poly-ICLC, is currently under investigation in a phase 1 clinical trial for pleural mesothelioma (NCT04525859).^32^ Given the changes observed in the antigen presenting cell populations with surgery exposure in our model, we hypothesized that PRR agonism targeting TLR3, which is predominantly expressed on dendritic cells, around the time of surgery would mitigate the pro-tumoral effect of surgery. We therefore tested perioperative systemic administration of poly(I:C) in our AGAS model (Fig. 4A). Perioperative poly(I:C) was capable of reversing AGAS, while it did not demonstrate an intrinsic antitumor effect in the control mice (Fig. 4B). Beyond reversing the early tumor growth effect, poly(I:C) also selectively reversed the surgery-induced effects on tumor-specific survival (Fig. 4C). Next, we tested the surgery-responsive splenic myeloid cell populations for changes following poly(I:C) administration compared to surgery alone. Interestingly, M-MDSCs or PMN-MDSCs did not show any alterations in systemic population density with the addition of poly(I:C) (Fig. 5A, B). In contrast, F4/80^+^ macrophages further decreased in frequency compared to surgery alone (Fig. 5C). Dendritic cells, however, increased in frequency with poly(I:C) administration, suggesting a central role for this cell population in mitigating the AGAS phenotype (Fig. 5D). Poly(I:C) did not affect frequency of T cell populations (Fig. S5). Absolute cell counts for the major myeloid and lymphoid parent populations in the context of poly(I:C) treatment are listed in Tables S6–S8.

## Discussion

The findings presented in this report demonstrate for the first time that the host response to the non-excisional components of abdominal surgery induces a systemic pro-tumoral effect in a mouse model of HR-NB. Our results reproduce and expand upon previously reported observations in other tumor types by applying a surgical stress model to one of the most common pediatric solid tumors. Importantly, the pro-tumoral surgery effect is eliminated when the model is tested in the broadly immune-deficient TKO mouse lacking T, B, and NK cells, strongly implicating a critical role for the immune system in the observed pro-tumoral surgery effect. As early as 5 d following non-excisional surgery, M-MDSCs and PMN-MDSCs are increased, while Mφ and DCs are decreased. Furthermore, perioperative administration of poly(I:C) effectively reverses the pro-tumoral surgery effect and further modulates the immune cellular changes induced by non-excisional surgical stress.

Long-standing evidence has shown that surgical resection of primary tumors leads to systemic immunosuppression, which in turn supports residual tumor outgrowth, recurrence and metastasis.^33^ However, the mechanistic contribution of the host response to the non-excisional components of surgery for tumor removal remains poorly understood. Surgery is an established modulator of several immune cellular populations including MDSC, DC, Mφ, NK cells, and T cells, with these changes contributing to post-operative systemic immunosuppression.^34^ In our model, we detected changes in systemic myeloid populations, including MDSC and antigen-presenting cells shortly after surgery. The myeloid changes were not accompanied by early systemic changes in the CD3^+^ lymphocytes or NK1.1^+^ NK cell populations in spleen, despite previous reports of surgery-induced changes within these populations in specific metastatic organ contexts.^30^ However, subtle changes in the CD4^+^ and CD8^+^ T cell populations were detected in TDLN even at 5 d after surgery exposure, earlier than would typically be expected for changes in lymphocyte populations. This observation suggests that surgery may be generating a change in trafficking signals for lymphocytes and regulating their movement in or around the TME. Our data raise a similar question for the myeloid populations changing with surgery: are these populations differentially trafficking after surgery exposure, or is surgery affecting the production of these populations overall? Investigating late timepoints after the surgical insult, as well as testing the TME itself, would be anticipated to more fully elucidate the mechanisms of immune cell responses to surgery exposure and will be the focus of future studies. Furthermore, the observed decrease in DC following surgery, coupled with the increase of DC by perioperative poly(I:C) therapy, suggests the potential for surgery to modulate the bridging of innate to adaptive antitumor immunity. Taken together, our data suggest a myeloid-mediated regulation of immune effector cell anti-tumor function after surgery.

Targeting the perioperative window offers unique therapeutic opportunities.^15^ Surgery for cancer is typically a planned event, scheduled at least several days and up to several weeks in advance. This advance knowledge of when the surgical stress will occur enables use of a preemptive, or a vaccination-like strategic approach to mitigate AGAS. We therefore hypothesized that a preemptive strategy would offer the maximal potential therapeutic benefit in our model when testing poly(I:C). When considering the translational potential of immune modifying agents, targeting the perioperative window offers the opportunity to test any agent with disease efficacy which may overcome some of the therapeutic barriers in neuroblastoma^35^ and other potential cancer types. Even agents that previously failed in therapeutic pre-clinical experiments or human clinical trials may be suitable for re-testing in the specific context of the perioperative window.

Innate immune activation is one therapeutic strategy that may mitigate unintended pro-tumorigenic effects of surgery, while minimizing potential negative impacts on wound healing and surgical recovery.^15^ For example, anticoagulants have been studied extensively and illustrated significant attenuation of lung metastases post-surgery.^36^ Toll-like receptors (TLRs), one kind of critical pattern recognition receptor (PRR) responsible for activating the innate immune system, are widely expressed on leukocytes and some non-immune cells.^37^ Experimental studies have shown that both TLR4 and TLR9 agonists are able to significantly ameliorate metastasis by activating NK cell cytotoxicity during the perioperative period without adverse effects.^38^^,^^39^ Poly(I:C) has demonstrated pre-clinical efficacy as an anti-cancer agent through indirect DC activation.^40^ Interestingly, in our model, perioperative administration of poly(I:C) reversed AGAS without altering tumor growth of the 9464D cell line in non-operated control mice. The susceptibility of this cell line to poly(I:C) has not previously been tested. Perioperative poly(I:C) also increased systemic DC frequency compared to the surgery-alone condition, though it did not affect T cell populations. Poly(I:C) is known to increase antigen cross-presentation by DCs through MHC-I/HLA-A2 pathways.^41^ The effect of poly(I:C) on AGAS in our model may be mediated through an MHC-I-based mechanism that regulates the functional interaction between DCs and CD8^+^ cytotoxic T cells. This interaction can be the focus of future investigations in other tumor models and across the age span to better understand the full potential of poly(I:C) as a perioperative therapeutic approach.

This work is the first to our knowledge to report AGAS in neuroblastoma. Our specific immune-competent AGAS model provides an excellent platform for understanding the impact of host responses to abdominal surgery and can be potentially applied to other tumor types, as we report for melanoma and colon adenocarcinoma. This model builds upon previous AGAS models that involve simulated metastatic disease via tail vein injection of tumor cells,^30^ combining the experimental surgical stress from those models with an established immune-competent model of *MYCN-*amplified HR-NB, which has been utilized to investigate combined immunotherapy in HR-NB.^25^ Our AGAS model enables specific delineation of the host response to surgery alone on distant disease outside the surgical field, independent of the effects arising directly from primary tumor removal. Though clinical cancer surgery inherently involves both the host response to surgery and primary tumor removal, the ability of our AGAS model to isolate the host response broadens the scope of future therapeutic discovery. Specifically, this model allows for exploration beyond tumor-intrinsic mechanisms of growth, metastasis, and treatment resistance.

In conclusion, we report an immune-mediated AGAS model in HR-NB that isolates the impact of the host response to surgery on distant disease located outside the surgical field. Perioperative administration of poly(I:C) selectively reverses the AGAS effect in our model, highlighting the potential to therapeutically target AGAS. Surgery in the AGAS model regulates multiple systemic immune cell populations, and poly(I:C) partially reverses these immune cellular changes. This HR-NB AGAS model is readily applicable for studying the impact of abdominal surgery on other tumor types and serves as a robust discovery platform for future research aimed at understanding the mechanisms driving AGAS and identifying therapeutic targets to improve outcomes for patients undergoing cancer surgery.

## Supplementary Material

vlaf058_Supplementary_Data

## Data Availability

Data relevant to the study are included in the article or uploaded as online supplemental information. Any further information about resources and reagents or the data underlying this article will be shared on reasonable request to the corresponding author.
