# Supplementary material for: Perioperative poly(I:C) reverses accelerated tumor growth after surgery in neuroblastoma
Source: Immunohorizons. 2025 Oct 9;9(11):vlaf058. doi: 10.1093/immhor/vlaf058 (PMC12597879; doi:10.1093/immhor/vlaf058)
Supplement: vlaf058_Supplementary_Data [file vlaf058_supplementary_data.pdf]

Supplemental Figure 1

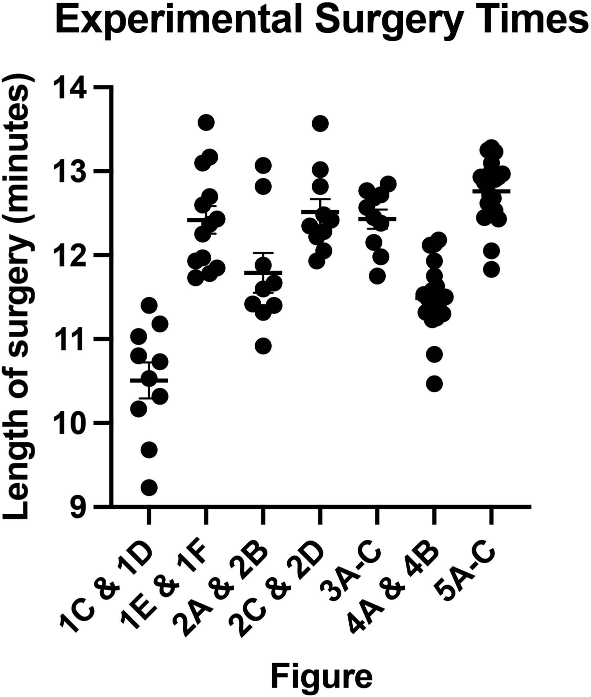

Supplemental Figure 2

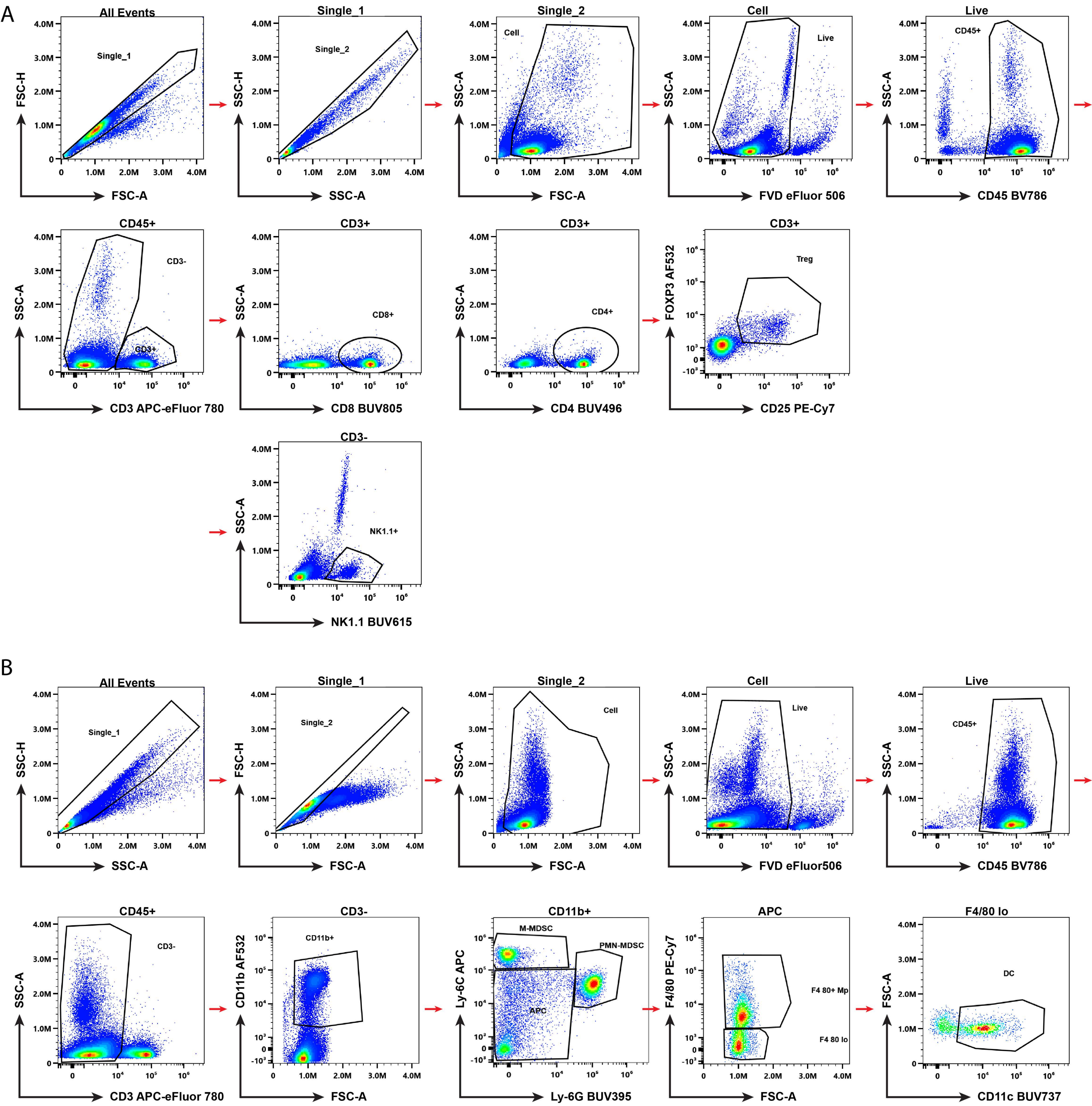

Supplemental Figure 3

A

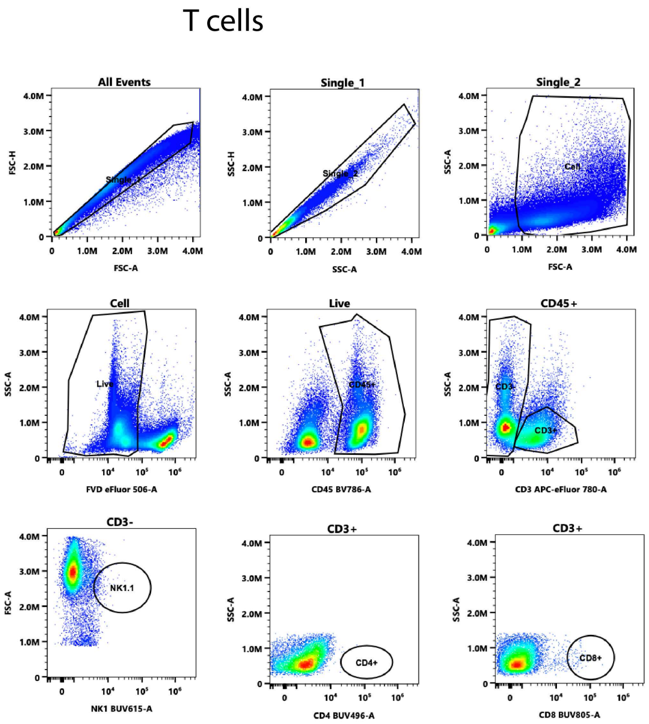

B

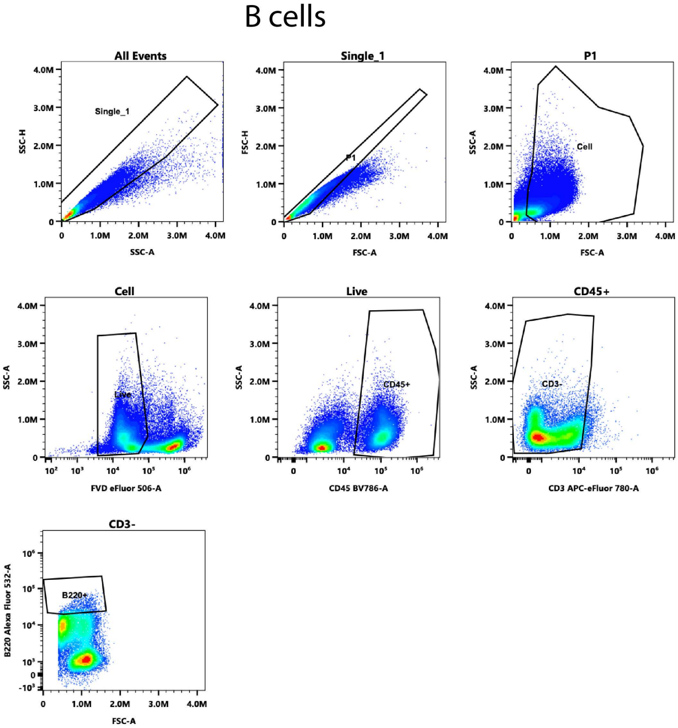

Supplemental Figure 4

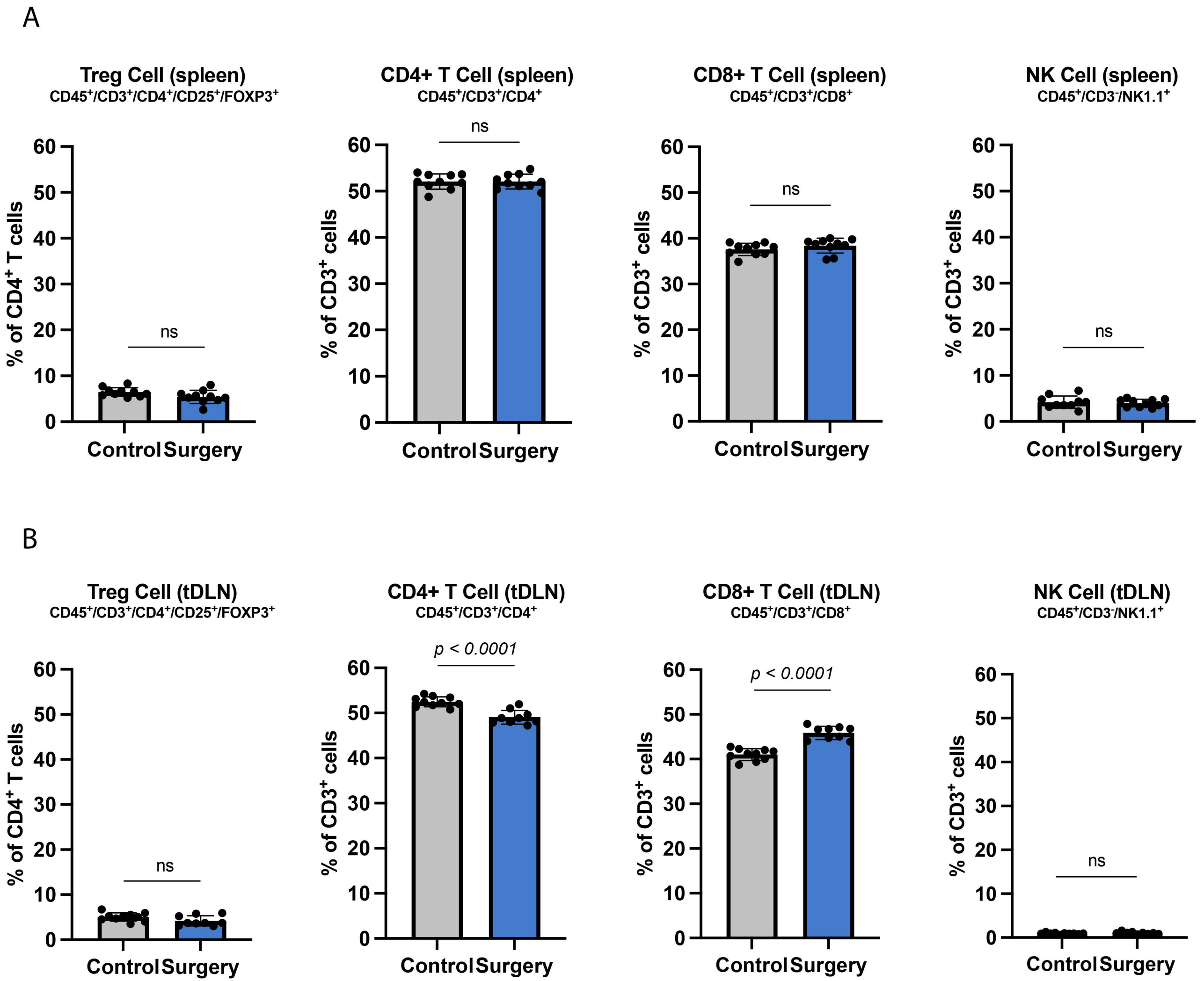

Supplemental Figure 5

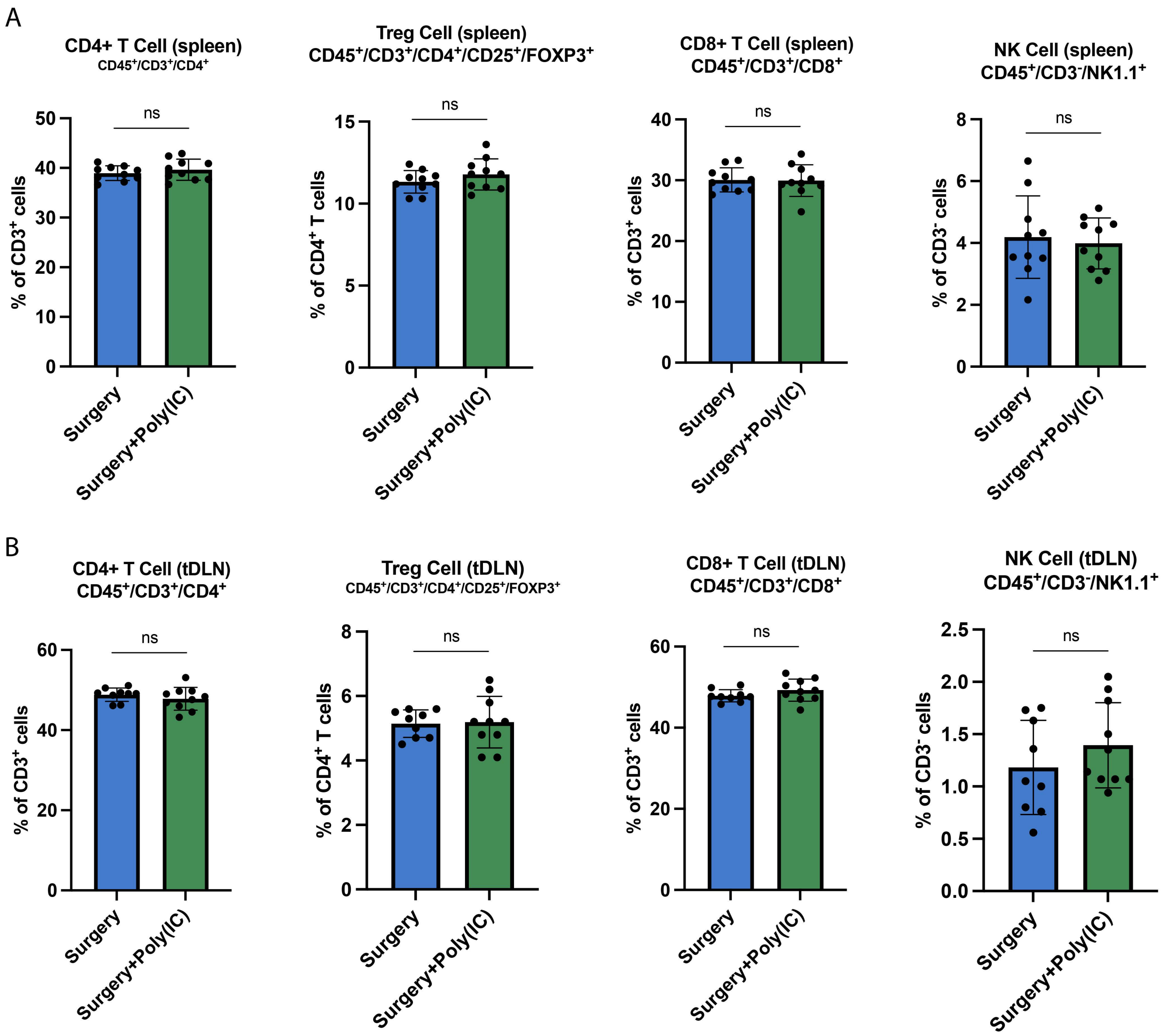

Supplemental Table 1. Antibodies for flow cytometry.

Target Antigen	Fluorochrome	Clone	Host isotype
CD16/32		S17011E	Rat
FVD	eF506	65-0866-14	
CD3ε	APC-eFluor 780	145-2C11	Rat
CD45	BV786	30-F11	Rat
CD25	PE-Cy7	PC61.5	Rat
CD4	BUV496	GK1.5	Rat
NK1.1	BUV615	PK136	Rat
CD8α	BUV805	53-6.6	Rat
FoxP3	Alexa Fluor 532	FJK-16s	Rat
Ly6C	APC	HK1.4	Rat
F4/80	PE-Cy7	BM8	Rat
Ly6G	BUV395	1A8	Rat
CD11c	BUV737	HL3	Hamster
CD11b	Alexa Fluor 532	M1/70	

Supplemental Table 2. Myeloid cell counts for spleen.

	**Sample**	**CD45+**	**CD3-**	**CD11b+**
Control	1	100758	63120	9414
	2	101296	54415	10796
	3	101190	63565	8506
	4	101268	66451	8945
	5	101182	66210	8266
	6	101211	59639	9903
	7	100773	56550	12511
	8	84281	55247	7372
	9	101092	63727	7664
	10	101356	63871	8690
	**Average**	**99441**	**61280**	**9207**
	**Sample**	**CD45+**	**CD3-**	**CD11b+**
Surgery	1	101784	66030	11788
	2	100476	65634	11822
	3	103673	64616	12145
	4	100670	67126	13897
	5	100676	65566	13606
	6	100704	69346	21283
	7	100703	68277	19821
	8	100587	61679	17514
	9	100740	63346	14769
	10	101290	65716	11120
	Average	**101130**	**65734**	**14777**

Supplemental Table 3. Myeloid cell counts for TDLN.

Sample	Total	CD45+	CD3-	CD11b+
1	57096	25416	3486	286
2	54752	22869	2733	341
3	55720	26910	3694	300
4	54336	25711	4534	349
5	58216	26387	4542	295
6	57192	25303	3582	353
7	42344	15988	2226	221
8	64920	28348	6646	411
9	58568	26715	5941	415
10	90005	35520	8370	476
**Average**	**59315**	**25917**	**4575**	**345**
**Sample**		**CD45+**	**CD3-**	**CD11b+**
1	14336	5114	812	160
2	84720	41330	10938	796
3	86408	42719	7147	778
4	47464	18128	1801	355
5	56800	26722	5379	364
6	60968	30586	5895	516
7	58224	27119	5171	506
8	60448	31609	5207	461
9	60952	30478	7015	374
10	62224	28435	7652	753
Average	**59254**	**28224**	**5702**	**506**

Supplemental Table 4. Lymphoid cell counts for spleen.

	Sample	CD45+	CD3+
Control	1	99409	35810
	2	99483	44040
	3	99482	36594
	4	99565	32606
	5	99710	33710
	6	99603	39293
	7	99729	41295
	8	99691	32556
	9	99691	32909
	10	99671	33770
	**Average**	**99603**	**36258**
	**Sample**	**CD45+**	**CD3+**
Surgery	1	99412	32280
	2	99632	33848
	3	99636	34814
	4	99651	33039
	5	99606	33373
	6	99505	31114
	7	99565	31422
	8	99682	35652
	9	99654	33230
	10	99565	29771
	**Average**	**99591**	**32854**

Supplemental Table 5. Lymphoid cell counts for TDLN.

	Sample	CD45+	CD3+
Control	1	101033	66666
	2	101150	60325
	3	100920	57702
	4	100938	66362
	5	100981	60983
	6	101045	64534
	7	114782	60454
	8	107386	58670
	9	103214	64718
	10	101975	69248
	**Average**	**103342**	**62966**
	**Sample**	**CD45+**	**CD3+**
Surgery	1	100840	67098
	2	100785	59641
	3	100852	60422
	4	100908	62106
	5	100990	59650
	6	100921	57227
	7	100920	57816
	8	100774	62246
	9	100864	68423
	Average	**100873**	**61625**

Supplemental Table 6. Myeloid cell counts for poly(I:C)-treated spleen.

	Sample	CD45+	CD3-	CD11b+
Surgery	1	102999	65615	11535
	2	102878	60455	11863
	3	103269	62849	11003
	4	103114	59856	11630
	5	102119	71762	11826
	6	102924	67225	12866
	7	103567	67378	17409
	8	103448	66337	15421
	9	103330	69549	12310
	10	102475	69888	13472
	**Average**	**103012**	**66091**	**12934**
	**Sample**	**CD45+**	**CD3-**	**CD11b+**
Surgery + Poly IC	1	101968	57394	9044
	2	101719	61483	6375
	3	101930	61847	15098
	4	102324	62299	10122
	5	102198	62691	16642
	6	103007	63084	18349
	7	102540	63497	10090
	8	102463	63910	15336
	9	102581	61425	12480
	10	102165	66965	10009
	**Average**	**102290**	**62460**	**12355**

Supplemental Table 7. Lymphoid cell counts for poly(I:C)-treated spleen.

	Sample	CD45+	CD3+
Surgery	1	101548	33819
	2	101334	40919
	3	101473	39451
	4	101366	41604
	5	101018	34295
	6	100612	39148
	7	101771	36489
	8	101188	35464
	9	101346	34269
	10	101361	36992
	**Average**	**101302**	**37245**
	**Sample**	**CD45+**	**CD3+**
Surgery +Poly IC	1	100895	36506
	2	100942	35434
	3	101725	35578
	4	101338	32320
	5	101663	34685
	6	101513	33819
	7	101289	32342
	8	101562	33201
	9	101157	31976
	10	101130	31081
	**Average**	**101321**	**33694**

Supplemental Table 8. Lymphoid cell counts for poly(I:C)-treated TDLN.

	Sample	CD45+	CD3+
Surgery	1	75557	44876
	2	76454	48020
	3	75934	44465
	4	76108	45102
	5	76041	43330
	6	76812	47254
	7	77007	42728
	8	76029	48178
	9	75805	40511
	**Average**	**76194**	**44940**
	**Sample**	**CD45+**	**CD3+**
Surgery + Poly IC	1	75542	47236
	2	100587	60268
	3	76027	39832
	4	76080	40592
	5	75867	40624
	6	76104	42576
	7	75649	40360
	8	75874	41098
	9	75542	47230
	10	76257	42640
	**Average**	**78665**	**43913**
